# Expression of Intelectin-1, also known as Omentin-1, is related to clinical phenotypes such as overweight, obesity, insulin resistance, and changes after bariatric surgery

**DOI:** 10.1038/s41598-024-72720-5

**Published:** 2024-09-27

**Authors:** Paul Czechowski, Tobias Hagemann, Adhideb Ghosh, Wenfei Sun, Hua Dong, Falko Noé, Corinna Niersmann, Isabel Reinisch, Christian Wolfrum, Christian Herder, Arne Dietrich, Matthias Blüher, Anne Hoffmann

**Affiliations:** 1 Helmholtz Institute for Metabolic, Obesity and Vascular Research (HI-MAG) of the Helmholtz Zentrum München at the University of Leipzig and University Hospital Leipzig, Philipp-Rosenthal-Straße 27, 04103 Leipzig, Germany; 2 Institute of Food, Nutrition and Health, ETH Zurich, Schmelzbergstrasse 9, 8092 Zurich, Switzerland; 3 Deutsche Diabetes-Zentrum, Leibniz Center for Diabetes Research at Heinrich Heine University Düsseldorf, Institute for Clinical Diabetology, Auf’m Hennekamp 65, 40225 Düsseldorf, Germany; 4 Deutsches Zentrum für Diabetesforschung, Ingolstädter Landstraße 1, 85764 Oberschleißheim, Germany; 5 Department of Endocrinology and Diabetology, Medical Faculty and University Hospital Düsseldorf, Heinrich Heine University, Moorenstraße 5, 40225 Düsseldorf, Germany; 6 Clinic and Outpatient Department for Visceral, Transplantation, Thoracic, and Vascular Surgery, Leipzig University Hospital, Liebigstraße 20, Haus 4, 04103 Leipzig, Germany; 7 Department of Endocrinology, Nephrology, Rheumatology, Leipzig University Hospital, Liebigstraße 20, Haus 4, Leipzig, 04103 Germany

**Keywords:** Diagnostic markers, Metabolism, Fat metabolism, Risk factors, Obesity, Diabetes, Obesity

## Abstract

Intelectin-1 (*ITLN1;* also Omentin-1, *OMNT1*) is secreted by adipose tissue (AT) and plays an important role in glucose metabolism regulation, with links to obesity-associated diseases. *ITLN1* activity so far has rarely been investigated using RNA-sequencing and in larger cohorts. We evaluated *ITLN1* expression among three clinical cohorts of the Leipzig Obesity BioBank—a cross-sectional cohort comprising of 1480 people, a cohort of people with metabolically healthy or unhealthy obesity (31 insulin-sensitive, 42 insulin-resistant individuals with obesity), and a longitudinal two-step bariatric surgery cohort (n = 65). We hypothesized that AT *ITLN1* expression is associated with serum omentin-1, clinical parameters associated with obesity, and with weight loss after bariatric surgery. We also investigated the correlation of AT *ITLN1* expression with genes related to inflammatory response, lipid metabolism, obesity, and regulation of energy balance. Likewise, we inspected gene group expression and metabolic pathways associated with *ITLN1* expression using gene set enrichment and gene correlation analysis. We show that *ITLN1* expression differs in VAT and SAT, and should therefore be analyzed separately. Furthermore, *ITLN1* expression increases with VAT tissue mass, but is negatively affected by AT tissue dysfunction among individuals with unhealthy obesity, corroborated by interplay with genes related to tissue inflammation. Gene set enrichment and gene correlation analysis of *ITLN1* expression suggest that AT *ITLN1* expression is related to local inflammatory processes in AT, but also in processes such as regulation of appetite, energy balance, and maintenance of body weight.

## Introduction

Obesity and overweight are major health concerns. About 1.9 billion people are affected by overweight worldwide, and 650 million by obesity; among whom are many children^1,2^. Obesity and overweight are characterized by excessive accumulation of adipose tissue (AT) that is linked to risk of hypertension, type 2 diabetes, coronary heart disease, stroke, gallbladder disease, osteoarthritis, sleep apnea and breathing problems, various cancers, and a low quality of life, with mental illness and physical impairment^1,2^.

AT stores energy and serves as an endocrine organ that secretes peptides (adipokines) and releases metabolites, including lipids and exosomal microRNAs^3,4^. Adipokines produced by AT include leptin, adiponectin, resistin, apelin, and also an isoform of omentin, omentin-1 (*OMNT1*), also known as intelectin-1 *(ITLN1)*^3,5,6^. The expression and secretion of adipokines partly depends on the fat depot source including visceral AT (henceforth: VAT) and subcutaneous AT (henceforth: SAT).

*ITLN1* is expressed in many tissues^3^, but was initially discovered being secreted from VAT, and has been associated with insulin action^7^. ITLN1 plasma levels are decreased in individuals with obesity^8^, and since its discovery, *ITLN1* has been primarily associated with diseases related to obesity^9–11^. In addition to regulating insulin action, ITLN1 has been found to have anti-inflammatory, antioxidative, anti-apoptotic, and anti-microbial effects, while possibly contributing towards preventing cardiovascular disease^3,4,12^. Consequently, ITLN1 is considered as a potential biomarker for prevalent metabolic diseases and considered a highly interesting therapeutic target^3^.

RNA sequencing is a useful high throughput transcriptome analysis tool to elucidate the relationship between gene expression signatures and clinical phenotypes, particularly when only small amounts of RNA extract are available^13,14^. So far, the relationship between ITLN1 and clinical parameters appears to have been investigated mainly using serum—or plasma—level studies, RT-PCR of cDNA, Western blot analysis, or analysis of expressed sequence tags; often coupled with relatively small study groups (but see one a large-cohort serum-level study^11^). To further investigate the relationship between ITLN1 expression levels and clinical obesity markers, we studied the gene expression of *ITLN1* in SC and VAT across three clinical subcohorts of the Leipzig Obesity BioBank (LOBB). We analyzed data of a cross-sectional cohort (CSC) comprising 1480 persons, a cohort of 73 persons with either metabolically healthy (MHO, n = 31) or unhealthy obesity (MUO, n = 42), and a cohort of 65 persons who underwent bariatric surgery (bariatric surgery cohort, BSC) for weight reduction (See methods and for CSC: Suppl. Table 1, MHO/MUO: Suppl. Table 2, BSC: Suppl. Table 3). Motivated by previous transcriptome analyses not supported by RNA sequencing^3,7,8,15–19^, we initially established whether *ITLN1* expression correlates to ITLN1 serum levels, and whether expression differed, among VAT or SAT in our RNA sequencing data, across all cohorts. We then investigated the association of obesity status, sex, insulin resistance, and weight reduction (after bariatric surgery) on *ITLN1* expression. Lastly, through Gene Set Enrichment Analysis (GSEA^20^), we elucidated statistically significant associations of *ITLN1* expression with co-expressed genes, associated gene pathways, and biological processes related to obesity. We discuss our findings in the context of genes known to be affected by ITLN1^21^.

## Results

### *ITLN1* expression is higher in VAT compared to SAT and positively correlates with ITLN1 serum concentrations

**Fig. 1:**
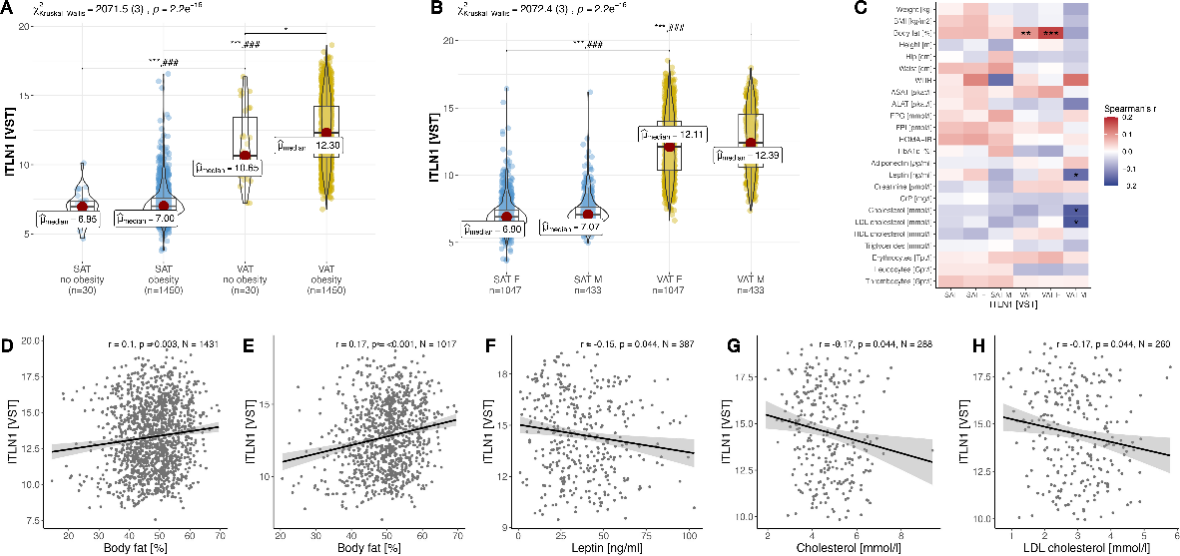
Analysis of *ITLN1* gene expression in the LOBB cross-sectional cohort (CSC). (**A**) Comparison of *ITLN1* expression between patients with and without obesity for subcutaneous and visceral adipose tissue (VAT and SAT respectively). (**B**) Analogous comparison for females and males. (**C**) Correlation of *ITLN1* gene expression with clinical parameters including all CSC patients, separately for females (F), and separately for males (M). (**D**) Scatter plot of the correlation of body fat with *ITLN1* expression measured VAT for all probands, and (**E**) in VAT for females. Shown in (**F**) is correlation of males’ VAT *ITLN1* expression with serum leptin, (**G**) Cholesterol, and (**H**) LDL-Cholsterol. We used Kruskal–Wallis one-way ANOVAs and the Mann–Whitney U test for pairwise comparisons among violin plots and corrected for multiple comparisons using the Hommel method. Correlations in boxplots are FDR-adjusted Spearman’s correlation coefficients. *P*-value symbols and significance: *** *p* < 0.001; ** *p* < 0.01; * *p* < 0.05; ### comparisons with the other tissue groups are all significant (*p* < 0.001).

**Fig. 2:**
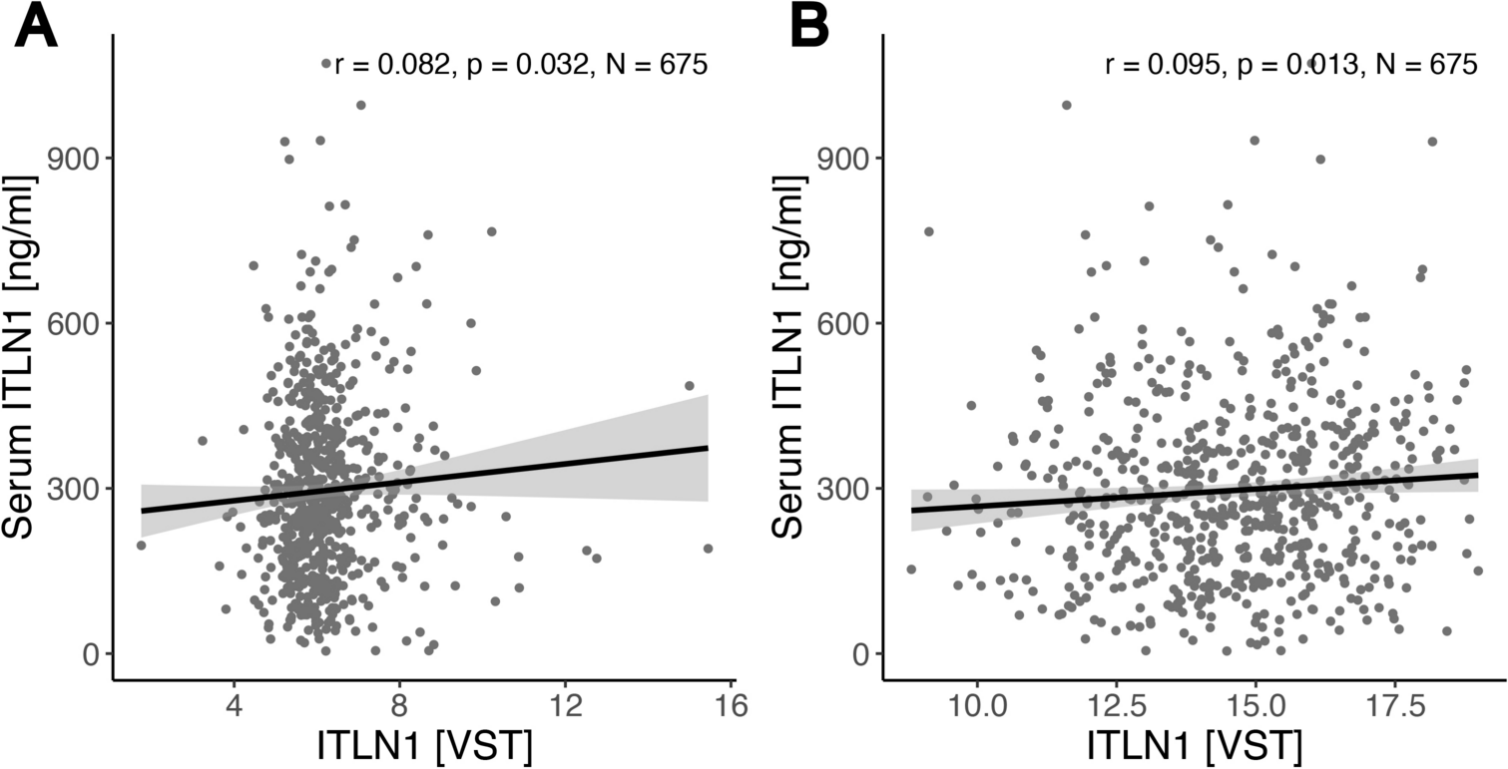
*ITLN1* gene expression and ITLN1 serum levels within the cross-sectional cohort (CSC). Shown are Spearman correlations in (**A**) subcutaneous fat, and (**B**) visceral fat. Shown are FDR-adjusted Spearman’s correlation coefficients.

**Fig. 3:**
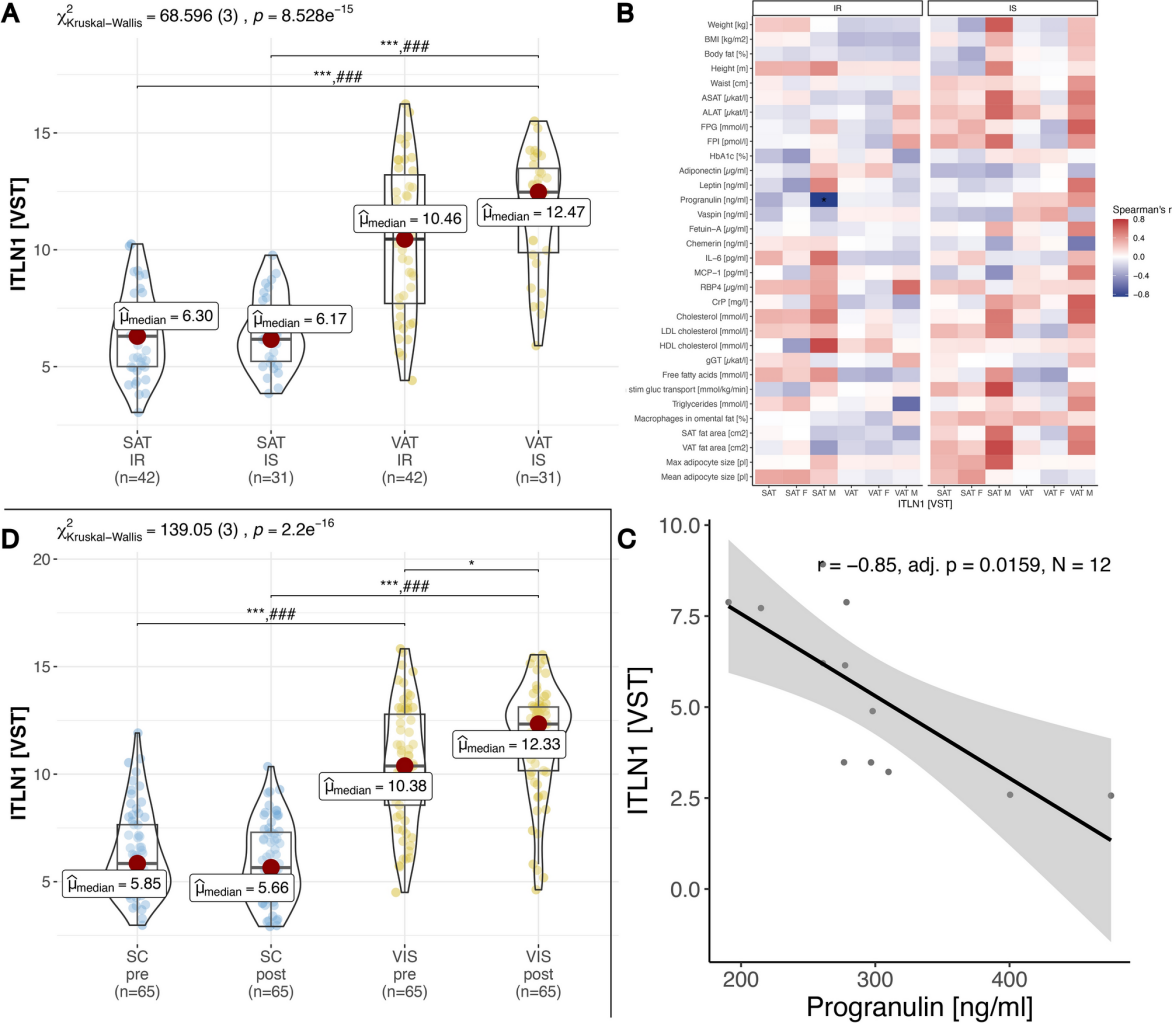
*ITLN1* gene expression among the MHO/MUO cohort, in people with metabolically healthy, insulin sensitive (IS) versus metabolically unhealthy, insulin resistant (IR) obesity. (**A**) Comparison of *ITLN1* expression between insulin resistant (IR) and insulin sensitive (IS) persons for SC and VAT. (**B**) Correlation of *ITLN1* gene expression in VIS and SAT with a comprehensive set of clinical parameters including all insulin-resistant (IR) or insulin-sensitive (IS) persons among the MHO/MUO as well as in female (F) and male (M) subsets. (**C**) Scatterplot of FDR-adjusted Spearman’s correlation between *ITLN1* expression and progranulin in VAT of IR males. Spearman’s correlation coefficient and FDR-adjusted for multiple testing. Inset (**D**) Analysis of *ITLN1* gene expression within the two-step bariatric surgery cohort (BSC). Comparison of *ITLN1* expression between the time point of first surgery and second step surgery after significant weight loss for SC and VAT. Kruskal–Wallis one-way ANOVA was performed, and the Mann–Whitney U test was used for pairwise comparisons, corrected for multiple comparisons using the Hommel method. *P*-value symbols and significance: *** *p* < 0.001; ** *p* < 0.01; * *p* < 0.05; ### comparisons with the other tissue groups are all significant (*p* < 0.001).

*ITLN1* expression significantly differed between VAT and SAT and were higher in VAT across all cohorts (CSC: Fig. 1A, MHO/MUO: Fig. 3A–C, BSC: Fig. 3D). Consequently, we conducted all subsequent analyses separately for either VAT or SAT. Among the available measurements (CSC), *ITLN1* gene expression and serum levels were positively correlated both in SAT (Fig. 2A, *ρ* = 0.08, n = 675, adj. *p* = 0.03), and in VAT (Fig. 2B, *ρ* = 0.1, n = 675, adj. *p* = 0.013).

### *ITLN1* expression is associated with obesity and exhibits sex-specific differences

*ITLN1* expression was higher in VAT of both women and men with obesity (Fig. 1B), and with increasing body fat in women. Mimicking this overall trend, *ITLN1* expression was higher in VAT then in SAT, regardless of obesity status (Fig. 1A). A higher *ITLN1* expression was found in people with obesity versus normal/overweight controls in VAT of both women and men with obesity (adj. *p* = 0.021, Fig. 1A). Among the CSC, VST-normalized and sex- and TIN-corrected median expression values in SAT were 6.95 for people with normal or overweight, and 7.00 for people with obesity (Fig. 1A). In VAT, median gene expression of *ITLN1* was 10.65 for people with normal or overweight and 12.30 for people with obesity (Fig. 1A).

Body fat mass positively correlates with *ITLN1* expression in VAT (ρ = 0.1, n = 1431, adj. *p* = 0.003; Fig. 1C,D), predominantly in women (ρ = 0.18, n = 1017, adj. *p* < 0.001; Fig. 1C,D,E). Among men, serum leptin levels (ρ =  − 0.15, n = 387, adj. *p* = 0.04; Fig. 1C,F) total cholesterol (ρ =  − 0.17, n = 288, adj. *p* = 0.04; Fig. 1C,G), LDL cholesterol (ρ =  − 0.17, n = 260, adj. *p* = 0.04; Fig. 1C,H; also see Suppl. Table 4) negatively correlate with *ITLN1* VAT expression.

Across several subdivided CSC data sets we also noted multiple insignificant (*p* < 0.05, but adj. *p* > 0.05) positive or negative correlations between ITLN1 expression and other clinical variables (ALAT, ASAT, BMI, body fat, total cholesterol, erythrocytes, FPI, HbA1c, HOMA-IR, and leucocytes), as detailed in Suppl. Table 4.

### *ITLN1* expression is associated with parameters of insulin resistance

In the MHO/MUO cohort, insulin-sensitive persons exhibited a trend towards higher *ITLN1* expression only in VAT, when compared to IR persons (Fig. 3A). In men with insulin resistance, (after FDR correction only) progranulin levels negatively correlated with *ITLN1* SAT expression (ρ =  − 0.85, n = 12, adj. *p* = 0.016; Fig. 3B,C and Suppl. Table 5). Correlations between *ITLN1* expression and other variables (total cholesterol, HDL cholesterol, free fatty acids, HbA1c, height, IL-6, insulin stimulated glucose transport, leptin, mean adipocyte size, progranulin, triglycerides, visceral fat area; Fig. 3B and Suppl. Table 5) were not statistically significant after FDR correction.

### *ITLN1* expression is not significantly affected by bariatric surgery induced weight loss.

Among the BSC we found significantly higher *ITLN1* expression in VAT after bariatric surgery induced weight loss (Fig. 3D). Among the BSC cohort in both sexes, we could not detect further significant correlations between *ITLN1* expression on measured clinical variables, neither pre- or post-surgery, nor among the delta between measured variables between pre- to post surgery (Suppl. Table 6). Insignificant correlations (*p* < 0.05) between *ITLN1* expression and other variables measured among the BSC include ALAT, BMI, body fat, CRP, erythrocytes, HbA1c, height, hip circumference, HOMA-IR, leptin, thrombocytes, waist circumference, and body weight (Suppl. Table 6).

### *ITLN1* expression is linked to gene groups affecting smell perception, metabolic pathways, and brain activity

**Fig. 4:**
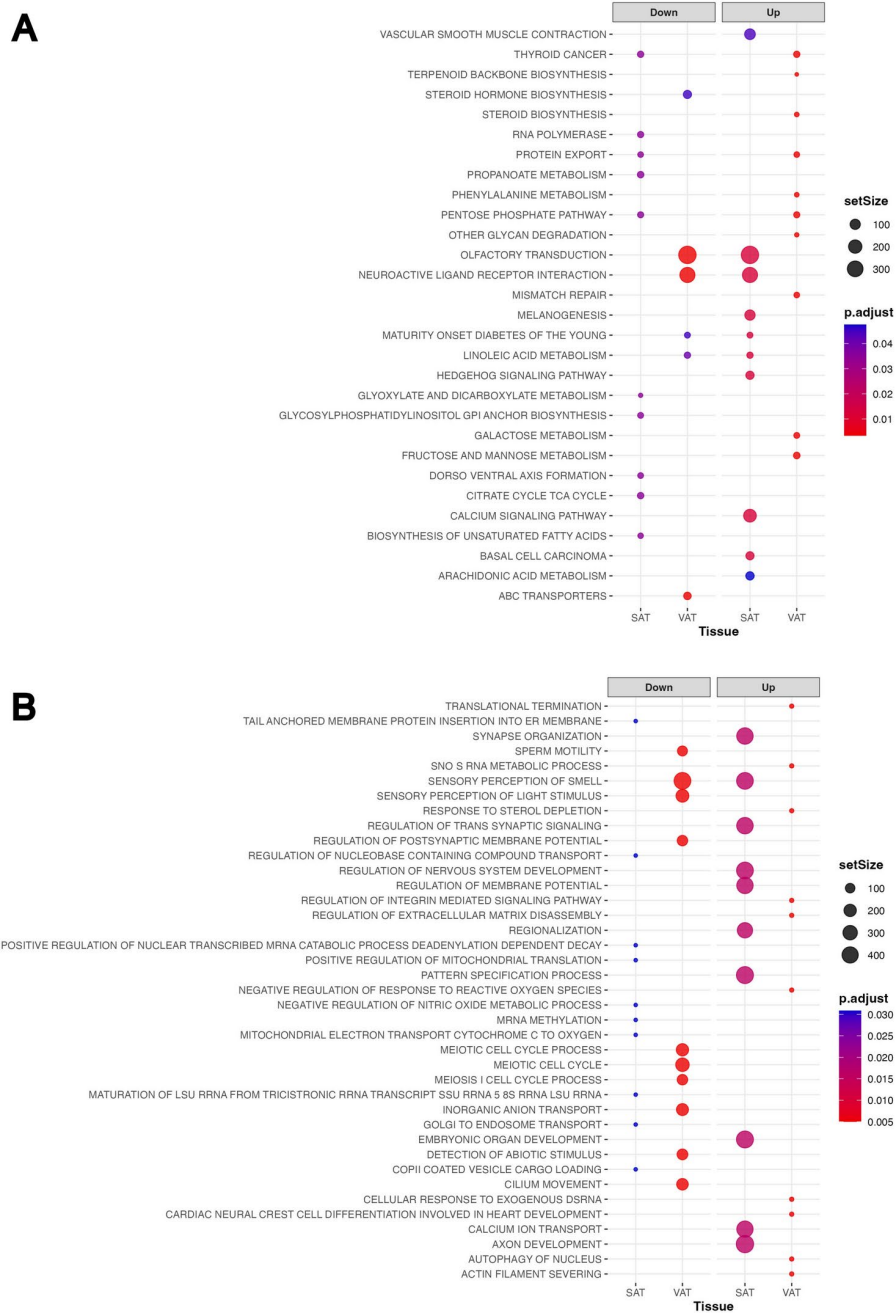
Results of Cross-sectional cohort (CSC) KEGG pathways and GO terms analyses. Among KEGG pathways (**A**) *ITLN1* expression appeared most significantly associated with downregulation of genes related to olfactory transduction in VAT (384 genes, normalized enrichment group score -2.808, adj. *p* 0.003) and their upregulation in SAT, (384 genes, norm. enrich. score 1.45, adj. *p* 0.013). In VAT the second-ranked significant expression association included downregulation of neuroactive ligand-receptor interactions (with 272 genes, norm. enrich. score − 2.08, adj. *p* 0.003), conversely upregulated in SAT (272 genes, norm. enrich. score 2.01, adj. *p* 0.013). Further information in Suppl. Table 4. (**B**) Among 4483 significantly enriched GO terms, the most significantly downregulated gene group in VAT (431 genes, norm. enrich. score − 2.91, adj. *p* 0.01) indicated expression patterns related to the sensory perception of smell. Most significantly enriched in SC was a gene group associated with axon development (474 genes, norm. enrich. score 1.48, adj. *p* 0.02. The second ranked GO-term group in VIS related *ITLN1* expression to downregulation of genes involved in the meiotic cells cycle (252 genes, norm. enrich. score − 1.8, adj. *p* 0.005). Other downregulated gene groups in VAT also related to meiotic cell cycle processes (e.g., 195 other genes, norm. enrich. score − 1.8, adj. *p* 0.005). The second-ranked most significantly enriched gene group in SAT related *ITLN* expression to upregulation synapse organization (414 genes, norm. enrich. score 0.0005, adj. *p* 0.02). Further information is provided in Suppl. Tab. 5.

In the CSC, analysis of 215 significantly enriched KEGG terms, representing gene groups either up- or down-regulated in VAT or SAT alongside *ITLN1* (see Suppl. Table 7) indicated *ITLN1* expression to be most significantly associated (Fig. 4A) with downregulation of genes related to olfactory transduction in VAT (384 genes, normalized enrichment group score − 2.808, adj. *p* 0.003), and their upregulation in SAT (384 genes, norm. enrich. score 1.45, adj. *p* 0.013). In VAT, the second-ranked significant expression association included downregulation of neuroactive ligand-receptor interactions (with 272 genes, norm. enrich. score − 2.08, adj. *p* 0.003), conversely upregulated in SAT (272 genes, norm. enrich. score 2.01, adj. *p* 0.013).

We also obtained 4483 significantly enriched GO terms, representing gene groups either up- or down-regulated in VAT or SAT alongside *ITLN1* (see Suppl. Table 8). The most significantly downregulated gene group in VAT (431 genes, norm. enrich. score − 2.91, adj. *p* 0.005; Fig. 4B) indicated expression patterns analogous to those obtained through KEGG analysis, related to the sensory perception of smell. Most significantly enriched in SC was a gene group associated with axon development (474 genes, norm. enrich. score 1.48, adj. *p* 0.02; Fig. 4B). The second ranked GO-term group in VAT related *ITLN1* expression to downregulation of genes involved in the meiotic cells cycle (252 genes, norm. enrich. score − 1.8, adj. *p* 0.005; Fig. 4B). Other downregulated gene groups in VAT also related to such meiotic cell cycle processes (e.g., 195 other genes, norm. enrich. score − 1.8, adj. *p* 0.005; Suppl. Table 5). The second-ranked most significantly enriched gene group in SAT related *ITLN*-expression to upregulation synapse organization (414 genes, norm. enrich. score 0.0005, adj. *p* 0.016).

Among the KEGG terms that were less significantly enriched, we observed the biosynthesis of unsaturated fatty acids in SAT (Fig. 4A), which are associated with genes of the beta-oxidation pathway, such as peroxisomal acyl-coenzyme A oxidase 1 *(ACOX1;* see Suppl. Table 7*)*. Additionally, we observed both up- and downregulation of genes related to maturity onset diabetes of the young (MODY) in VAT and SAT, respectively, such as hepatocyte nuclear factor *(HNF)* genes and Neurogenin 3 (*NEUROG3*; also see Suppl. Table 7).

Products of 19 genes associated with ITLN1 expression^21^ were confirmed to be predominantly involved in lipid metabolism, as well as proinflammatory and inflammatory process^22^ (see supplementary materials for detailed descriptions). Among the CSC, *ITLN1* expression in SAT correlated strongly negatively with *ICAM1* (encoding Intercellular Adhesion Molecule 1), *CCL2* (encoding C–C Motif Chemokine Ligand 2) and *PPARG* (Peroxisome Proliferator Activated Receptor Gamma), and strongly positively correlated with *MAPK13* (Mitogen-Activated Protein Kinase 13), *CARTPT* (encoding a prepropeptide of the Cocaine—And Amphetamine-Regulated Transcript Protein), and *CRH* (Corticotropin Releasing Hormone)—for all values see Table 1. In VAT, ITLN1 expression correlated strongly negatively with *NLRP3* (NLR Family Pyrin Domain 3), *PPARD* (Peroxisome Proliferator Activated Receptor Delta) and *MAPK13,* and strongly positively correlated with *CCL2*, *TXNIP* (Thioredoxin Interacting Protein) and PPARG—for all values see Table 2, correlations are presented graphically also in Suppl. Figs. 1 and 2, for SAT and VAT, respectively.

**Table 1:** Correlation of VST normalized expression values between ITLN1 and 19 associated genes (reviewed in^21^) shown for subcutaneous adipose tissue (SAT) among the cross-sectional-cohort (CSC, n = 1480).

Genes	r	SE	*p*
CXCL8	− 0.10***	0.03	< 0.01
NLRP3	-0.05	0.03	0.08
IL6	− 0.09***	0.03	< 0.01
TNF	− 0.07*	0.03	0.01
PPARD	0.08**	0.03	< 0.01
PRKAA1	0.08**	0.03	< 0.01
PRKAA2	0.14***	0.03	< 0.01
PRKAB1	0.14***	0.03	< 0.01
ICAM1	− 0.22***	0.03	< 0.01
IL1B	− 0.04	0.03	0.15
CCL2	− 0.16***	0.03	< 0.01
MAPK11	0.06*	0.03	0.02
MAPK12	0.2***	0.03	< 0.01
MAPK13	0.22***	0.03	< 0.01
MAPK14	− 0.2***	0.03	< 0.01
TXNIP	− 0.18***	0.03	< 0.01
PPARG	− 0.18***	0.03	< 0.01
CARTPT	0.08**	0.03	< 0.01
CRH	0.23***	0.03	< 0.01

We report FDR corrected Spearman correlations (r), the standard error (SE), and statistical significance (likelihood of observation occurance under null hypothesis, *p*). Graphic presentation is provided in Suppl. Figure 1. Gene abbreviations are expanded in the methods section.

## Discussion

We investigate associations between AT *ITLN1 (Omentin-1,* OMNT1*)* expression and clinical parameters related to obesity and its comorbidities in three clinically well-phenotyped obesity cohorts. Likewise, we present baseline information on correlations between *ITLN1* expression and expression of genes related to (pro-) inflammatory processes, immune response, adipocyte differentiation, obesity, appetite, energy balance, maintenance of body weight^21^.

**Table 2:** Correlation of VST normalized expression values between ITLN1 and 19 associated genes (reviewed in^21^) shown for visceral adipose tissues (VAT) among the cross-sectional-cohort (CSC, n = 1480).

Genes	r	SE	*p*
CXCL8	− 0.17***	0.03	0.00
NLRP3	− 0.27***	0.03	< 0.01
IL6	− 0.02	0.03	0.46
TNF	− 0.18***	0.03	< 0.01
PPARD	− 0.26***	0.03	< 0.01
PRKAA1	− 0.31***	0.02	< 0.01
PRKAA2	− 0.42***	0.02	< 0.01
PRKAB1	-0.28***	0.03	< 0.01
ICAM1	0.39***	0.02	< 0.01
IL1B	− 0.02	0.03	0.46
CCL2	0.48***	0.02	< 0.01
MAPK11	0.02	0.03	0.41
MAPK12	− 0.06*	0.03	0.03
MAPK13	− 0.44***	0.02	< 0.01
MAPK14	0.5***	0.02	< 0.01
TXNIP	0.42***	0.02	< 0.01
PPARG	0.26***	0.03	< 0.01
CARTPT	− 0.32***	0.02	< 0.01
CRH	− 0.23***	0.03	< 0.01

We report FDR corrected Spearman correlations (r), the standard error (SE), and statistical significance (likelihood of observation occurance under null hypothesis, *p*). Graphic presentation is provided in Suppl. Figure 2. Gene abbreviations are detailed in the Methods section.

Our expression analysis identified a weak positive correlation between serum ITLN1 (Omentin-1) and *ITLN1* expression among people with obesity, despite the markedly different expression levels across adipose tissues, which we also observe and corroborate. To integrate findings related to *ITLN1* levels both from serum-level studies and expression analyses, we aimed to relate both analysis methods, which we deem challenging. Firstly, persons with obesity have more AT then non-obese persons, so expression may seem similar between obese and control persons, but ITLN1 production rate may differ nonetheless^15^. Furthermore, *ITLN1* is expressed in many tissues and cell types of the body, primarily by mesothelial cells, vascular smooth muscle cells and endothelial cells of VAT, but also in epicardial fat, small intestine, colon, thymus, ovary, testis, in intestinal Paneth cells, further in airway and intestinal goblet cells^23^. Noteworthy, ITLN1 serum concentrations may not always correlate with gene expression, at least in mice^24^. It was hence important to us to show a direct relationship between *ITLN1* expression levels and serum levels. We find a weak positive relationship between ITLN1 serum levels and AT *ITLN1* expression particularly in VAT (Fig. 2). Associations between SAT *ITLN1* expression with phenotype parameters are weaker and could be partly explained by lower overall expression intensity of *ITLN1* in SAT^15^.

*ITLN1* gene expression has been shown to be higher in VAT than in SAT due to differences in the total production rate of adipose tissue-derived factors^15^ (also^3,7,25^). We confirmed *ITLN1* expression to be higher in VAT than in SAT across all cohorts (CSC, BSC, MHO/MUO). Therefore, we corroborate that *ITLN1* expression should be explored separately for each tissue type, also when among RNA sequencing data^3,7,15^. RNA sequence analysis^13^ thus seems valuable to analyze *ITLN1* expression*,* and in consequence likely also other expression of adipokines.

Although the correlation between ITLN1 serum levels and VAT *ITLN1* expression is not strong, both are significantly associated with VAT *ITLN1* expression (Fig. 2A,B). At the same time, this relationship does not appear linearly related to body weight. ITLN1 serum levels and gene expression have been reported to be lower in people with obesity^8,26^, and higher in women compared to men^8^ (Fig. 1b), comparable to other adipokines, such as adiponectin^27^. For a given BMI, women have more body fat then men^28^. Additionally, men exhibit lower serum leptin levels at any given measure of obesity^29^ and higher levels of serum total cholesterol, when compare to females^30^.

Our results support the notion that serum ITLN1 concentrations are predominantly determined by VAT *ITLN1* expression^15^. Our data also suggest that higher *ITLN1* expression among people with obesity does not lead to ITLN1 serum levels found in lean persons, possibly due to a nonlinear rise of *ITLN1* expression with increasing fat mass. Congruent with our results, this effect would be less pronounced for women when compared to men, as women have more body fat. Arguably, for those reasons, also cholesterol and leptin serum levels are inversely related to ITLN1 levels in men, and consequently the correlation between *ITLN1* expression may appear positive with leptin than that with cholesterol (ρ =  − 0.15, and ρ =  − 0.17, Suppl. Table 4).

Obesity-related adipose tissue dysfunction appears to impair VAT *ITLN1* expression upregulation with higher BMI classes and higher degree of obesity. Initially, this appearance is supported by the inverse correlation of *ITLN1* expression, and genes related to the inflammatory response in VAT (e.g. *CXCL8, TNF, NLRP3*; see Table 2; see methods for unabbreviated gene names) Furthermore, the *ITLN1* expression increase among people with obesity in our work appears to plateau earlier among IR persons (Fig. 2a), then among IS persons. This suggests that described associations between ITLN1 and parameters of obesity and metabolic diseases are caused by obesity-related AT tissue dysfunction^16,17,31^ and may explain why our data contradict some previous reports^8,26^. In addition, men are likely to be more affected by VAT dysfunction^25^, possibly due to a less favorable ratio of body fat to lean mass, when compared to women.

Notably, among men from the CSC and MHO/MUO cohorts, progranulin—a circulating marker of AT dysfunction and inflammation—is inversely correlated with visceral AT *ITLN1* expression. This inverse correlation is supported by the inverse relationship between *ITLN1* and genes related to the proinflammatory response in SAT (e.g., CXCL8, IL6, CCL2, TNF; see Table 1) and also in VAT (e.g., CXCL8, TNF, NLRP3, but not CCL2; see Table 2, Supplemental Fig. 2), and may also reflect lower ITLN1 serum levels in men compared to women and implicate sex differences in AT function status^32^.

In SAT and VAT not all genes related to the inflammatory response were correlated with *ITLN1* expression in the same way. This finding is in line with observations that gene expression of fat depots are regulated differently based on their fat depot location^33^—some expression differences in can be expected between VIS and SAT, especially when the many other genes related to the inflammatory response appear expressed inversely to *ITLN1*. Moreover, the relationship between *ITLN1* expression and gene expressions reflecting inflammation does not allow to draw conclusions regarding the direction (activated or reduced inflammation) or establish a causality chain. Our data still suggest that *ITLN1* expression is co-regulated to several genes of inflammatory response. Whether altered *ITLN1* adipose tissue expression is the cause or consequence of heterogeneity in inflammatory gene expressions needs to be investigated in further studies.

Overall, increased *ITLN1* expression in VAT after bariatric surgery is in line with previous findings^18,19^. The observed increased expression is indicative of AT tissue health regeneration after surgery-induced weight loss, facilitating increases of *ITLN1* expression. As suggested by our correlation analyses, during AT regeneration rising ITLN1 expression may affect regulation of appetite, energy balance, and maintenance of body weight through the *CARTPT* gene^22^ and changes to regulation in adipocyte generation through downregulated PPARG (see Table 1).

Given the importance of VAT for *ITLN1* expression and secretion described across literature and corroborated above, we believe the interpretation of *ITLN1 related* gene group activity in VAT to be of highest importance. Our results support a pivotal link of *ITLN1* expression throughout the metabolism in line with studies related to other adipokines such as visfatin, leptin, and adiponectin.

Brain response to food odors has been deemed unassociated with BMI and obesity-related metabolic health measures^34^. Opposing this finding, higher baseline HOMA-IR or circulating baseline insulin levels were correlated with poorer olfactory performances^35^. Our work supports the second finding—we observe an interplay of genes related to the sensory perception of smell in VAT and SAT. We hypothesize that ITLN1 affects similar genes in the brain and olfactory organs as to the genes observed in AT by us. We believe our hypothesis to be supported by the up- and down-regulation of Cocaine- And Amphetamine-Regulated Transcript Protein expression (CARTPT) related to *ITLN1* expression in SAT and VAT (see Table 1 and 2) which affects appetite, energy balance, maintenance of body weight of the entire body^22^ not in AT alone.

Adipokines are known to affect neuroactive ligand-receptor interaction pathways, as demonstrated for the regulation of food intake in chicks by visfatin^36^. It is thus standing to reason that *ITLN1* expression is linked to regulation of systemic metabolic processes. The interpretation of all enriched gene groups is beyond the scope of this study, but we provide three potential lines of evidence for our assumption that ITLN has systemic effects on the entire metabolism by affecting expression of genes that are not only active in AT: among the 272 affected genes associated with *ITLN1* expression and KEGG-Term “neuroactive ligand-receptor interaction” were the thyroid stimulating hormone receptor gene *TSHR*, the galanin receptor gene *GALR1*, and the μ-opioid receptor gene *OPRM1* (Suppl. Table 7).

Defects of the thyroid stimulating hormone receptor gene *TSHR* are deemed causative of hypothyroidism^37^. Galanin receptor mRNAs, including *GALR1* transcripts, are expressed in the central and peripheral nervous system as well as in peripheral tissues, including the digestive system^38^. The μ-opioid receptor gene *OPRM1* in peripheral tissue has been linked to control of immunological functions^39^, but also reacts to endogenous opioid peptides and opioid analgesic agents, and its mutation has been related to the vulnerability to frustration^40^ . We hence provide clues that while AT *ITLN1* expression may downregulate inflammatory processes in in AT, *ITLN1* expression may also influence system-wide metabolic functions much like other adipokines and may possibly affect mood.

Lastly, receptors of adipokines are widely expressed in the brain, and leptin and adiponectin can cross the blood–brain barrier, while evidence for newly identified adipokines has been reported to be sparse^41^. Our description of a linkage between *ITLN1* expression to axon and synapse development hence may hint at ITLN1 also affecting brain states and health, like other adipokines, even if the respective regulatory pathways are here only observed in AT. They are likely comparable in other tissues. Again, our train of thought is supported by the expression changes of *CARTPT* in SAT and VAT (see Tables 1 and 2) which are significantly correlated to *ITLN1* expression, hinting at the possibly that *ITLN1* may affect regulatory networks influencing appetite, energy balance, and maintenance of body weight throughout the entire body^22^, not in AT alone.

It is readily recognized that adipokines expressed in AT influence metabolic processes. It is hence also conceivable that *ITLN1* expression influences genes involved in fatty acid synthesis, such as *ACOX1* which encodes the first enzyme of the fatty acid beta-oxidation pathway^42^, among other genes. Furthermore, we hypothesize that *ITLN1* expression may exert an influence on the coupling between glucose sensing and insulin secretion, as well as the maintenance of fully differentiated β-cell phenotypes, through the finely tuned differential expression of *HNF*s, *NEUROG3*, and other genes associated with MODY^43,44^, in different adipose tissues. The observed differential up- and downregulation of these genes in SAT and VAT may result in varying net concentrations of the expressed gene products, based on the volume or weight ratio of both AT types. For instance, an increase in VAT would lead to a net downregulation of the involved genes, while an increase in SAT tissue would have the opposite effect. However, these regulatory mechanisms remain speculative.

In summary, we provide analyses of RNA-sequencing data relating *ITLN1* expression to various clinical parameters in large cohorts. *ITLN1* expression is associated with the expression of genes implicated in (pro-)inflammatory response, lipid metabolism and energy balance. We show that *ITLN1* expression differs in VAT and SAT. Furthermore, we found that *ITLN1* expression increases with VAT tissue mass, but is affected negatively by increasing AT tissue dysfunction among individuals with unhealthy obesity. Gene set enrichment and gene correlation analysis of *ITLN1* expression suggest that AT *ITLN1* expression is related to local inflammatory processes in AT but may also play a role in processes related to the regulation of appetite, energy balance, and maintenance of body weight.

## Materials and methods

### Cohorts

The LOBB was established to help understand obesity and its associated diseases. Here, we analyzed clinical data in association with gene expression data derived from paired human samples of VAT and SAT of three distinct cohorts. We include a cross-sectional cohort (CSC) comprising 1480 persons, a cohort of 73 persons with either metabolically healthy (MHO, n = 31) or unhealthy obesity (MUO, n = 42), and a cohort of 65 persons who underwent bariatric surgery (bariatric surgery cohort, BSC) for weight reduction.

Samples analysed in this work had been collected between 2008 and 2018 during elective laparoscopic abdominal surgery as described previously^45,46^. Laboratory measurements of body composition and metabolic parameters had been obtained concomitantly^47,48^. Tissue samples were taken from adult men and women aged 18 and older who underwent elective abdominal surgery, and who consented to study participation. All study protocols have been approved by the Ethics committee of the University of Leipzig (363-10-13122010 and 017-12-230112). All participants gave written informed consent before taking part in the study. Notable exclusion criteria include chronic substance or alcohol misuse, smoking within twelve months preceding the surgery, acute inflammatory diseases, usage of medications directly impacting adipose tissue, end-stage malignant diseases, weight loss exceeding 3% in three months prior to surgery, uncontrolled thyroid disorder, and Cushing’s disease.

Among the 1480 persons belonging to the CSC, 30 had normal weight/overweight (53% women; 56.4 ± 13.3 years; Body Mass Index (BMI) 25.5 ± 2.6 kg/m^2^), and 1450 had obesity (71% women; 46.9 ± 11.7 years; BMI 49.2 ± 8.3 kg/m^2^). In the CSC, serum ITLN1 levels were available from 675 LOBB participants, seven people with normal/overweight and from 668 people with obesity.

The MHO/MUO cohort consists of 31 insulin-sensitive (IS) persons (71% female; 38.8 ± 11.1 years; BMI 45.9 ± 6.9 kg/m^2^; fasting plasma glucose (FPG): 5.2 ± 0.2 mmol/l; fasting plasma insulin (FPI): 27.9 ± 13.5 pmol/l) and 42 insulin-resistant (IR) persons (71 female; 47.2 ± 7.7 years; BMIs 47.3 ± 8.1 kg/m^2^; FPG: 5.7 ± 0.3 mmol/l; FPI: 113.7 ± 45.7 pmol/l).

The BSC cohort consists of 65 persons with morbid obesity (66% women; BMI > 40 kg/m^2^) who all underwent a complete two-step bariatric surgery strategy. Surgeries typically involved laparoscopic sleeve gastrectomy as first step, followed by a laparoscopic Roux-en-Y gastric bypass surgery. Data were collected prior to each surgery and VAT and SAT samples were obtained during the surgery. The average preoperative BMI and age of the cohort patients were 54.5 ± 9.3 kg/m^2^ and 44.1 ± 9.2 years, respectively. At the second surgery, patients had an average BMI of 40.9 ± 7.2 kg/m^2^ and an average age of 47.1 ± 9.9 years. The patients lost an average of 40.2 ± 21.2 kg between the two surgeries, and only those (i.e., 65) who lost more than five kilograms were included into the BSC. Time between surgeries was 3.0 ± 3.9 years. Preoperatively, type 2 diabetes (T2D) was diagnosed in 28 patients, postoperatively T2D was still present in 18 patients. All people received frequent and structured healthy diet recommendations^49^.

### Clinical variables

Parameters considered for this work include (in alphabetical order) serum concentrations of adiponectin (µg/ml), alanine transaminase (ALAT) (µkat/l), aspartate transaminase (ASAT) (µkat/l), body fat (%), body mass index (BMI) (kg/m^2^), C-reactive protein (CRP) (mg/l), fasting blood glucose (FPG) (mmol/l), glycated hemoglobin A1c (HbA1c) (%), fasting plasma insulin concentration (FPI) (pmol/l), high-density lipoprotein (HDL) cholesterol (mmol/l), height (m), leptin (ng/ml), low-density lipoprotein (LDL) cholesterol (mmol/l), total cholesterol (mmol/l), triglycerides (mmol/l), waist circumference (cm), and weight (kg). Only for the CSC, serum ITLN1 (ng/ml) was available. For the CSC and BSC we additionally evaluated creatinine (µmol/l), erythrocytes (Tpt/l), hip circumference (cm), homeostasis model assessment of insulin resistance (HOMA-IR), leucocytes (Gpt/l), thrombocytes (Gpt/l), and waist-to-hip ratio (WHR). For BSC and MHO/MUO, we also evaluated serum interleukin 6 (IL-6) (pg/ml) and non-esterified fatty acids (NEFA) (mmol/l). Only for the MHO/MUO, we evaluated chemerin (ng/ml), fetuin-A (µg/ml), glucose infusion rate during the steady state of a hyperinsulinemic-euglycemic clamp procedure (mmol/kg/min), macrophage number in VAT (%), maximum and mean adipocyte diameter (pl), monocyte chemoattractant protein-1 (MCP-1) (pg/ml), progranulin (ng/ml), retinol binding protein 4 (RBP4) (µg/ml), SAT and VAT fat area (cm^2^), visceral adipose tissue derived serine protease inhibitor (vaspin) (ng/ml), and γ-glutamyl transferase (gGT) (µkat/l). Data availability for each evaluated variable across the respective cohorts are summarized in supplementary materials (CSC: Suppl. Table 1, MHO/MUO: Suppl. Table 2, BSC: Suppl. Table 3).

### Bulk RNA sequencing and analysis

We chose to use RNA sequencing to study gene expression of our precious RNA extracts due to the superior data yield compared to other techniques^13,14^. We extracted RNA from SC and VAT and prepared rRNA-depleted RNA-seq data following the SMARTseq protocol^50,51^. RNA was enriched and reverse-transcribed using Oligo(dT) and TSO primers, followed by cDNA amplification with *in-silico*-tested PCR primers. Complementary DNA was then processed using a Nextera DNA Flex kit with Tn5 transposase. Single-end sequencing of all libraries was performed on a Novaseq 6000 instrument at the Functional Genomics Center in Zürich.

We used *Fastp* (v0.20.0^52^) with a minimum read length of 18 nucleotides and a quality cut-off of 20 for raw sequencing read preprocessing, then *Kallisto* (v 0.48^53^) for read alignment against the human reference genome (assembly GRCh38.p13, GENCODE release 32) and gene-level expression quantification. All subsequent analyses were performed in R version 4.2.2^54^. Normalization to homoscedastic library sizes was achieved with variance stabilizing transformation (VST^55^) of raw transcript counts as implemented in *DESeq2* (v1.32.0^56^). To avoid tissue variation in gene expression for downstream correlation and Gene Set Enrichment Analysis (GSEA^20^), we normalized data of each tissue separately as *ITLN1* expression was significantly different between both tissues (Fig. 1a). Where required by excessive read counts, samples were down-sampled to 20 million reads with R package *ezRun* (v3.14.1^57^). Mitigating the effects of *in-vitro* RNA degradation, we calibrated normalized counts using transcript integrity numbers (TINs; estimated with R package *RSeQC* v4.0.0^58^). Despite large age differences between few cohort members, we did not detect age-related batch effects and therefore adjustment for age was deemed unnecessary. Batch effects were adjusted with *limma* (v3.56.2^59^) after investigation with R package *swamp* (v1.5.1^60^).

### Statistical analyses

Prior to exploring expression level differences between groups and clinical variables, we used one-way Kruskal–Wallis ANOVA and the Mann–Whitney U test for pairwise comparisons, corrected for multiple comparisons using the Hommel method^61^, implemented in R package *ggstatsplot* (v0.10.1^62^). We analyzed univariate Spearman correlations between *ITLN1* expression and clinical variables using R package *RVAideMemoire* (v0.9–81-2^63^) and corrected for multiple inferences by considering the sample-size-appropriate false discovery rate (FDR). We considered absolute correlations ρ ≥ 0.1 and adj. *p* < 0.05 to be relevant.

### Gene set enrichment analyses

GSEA can provide information on biological processes, such as metabolic pathways, transcriptional programs, and stress responses, which may be distributed across an entire network of genes, and thus hard to discover when inspecting individual genes^20^. Here, we used the R package *CorrelationAnalyzeR* (v1.0.0^64^), to interpret such gene groups, and their function, which are up- or downregulated alongside *ITLN1*. We aimed to determine whether statistically significant associations exist between *ITLN1* expression and pathways or biological processes, as defined by the Kyoto Encyclopedia of Genes and Genomes (KEGG)^65^, or by Gene Ontology (GO)^66^ terms. Specifically, we obtained GSEA annotations correlating with *ITLN1* expression by considering the CSC’s expression data from for VAT and SAT and employing *CorrelationAnalyzeR’s* single gene mode. The tool uses genome-wide Pearson correlations as a pre-ranking metric for the GSEA algorithm to determine gene set correlations with a gene of interest. Analyses were corrected for multiple testing by considering the FDR.

### Correlation of ITLN1 expression with associated genes

We correlated *ITLN1* expression values with 19 genes known to be associated with *ITLN1* expression, as recently reviewed^21^. These genes included C-X-C Motif Chemokine Ligand *(CXCL8)*, Interleukin 6 *(IL6)*, Interleukin 1 Beta *(IL1B)*, C–C Motif Chemokine Ligand 2 *(CCL2)*, Tumor Necrosis Factor *(TNF)*, Peroxisome Proliferator Activated Receptor Delta *(PPARD)*, Protein Kinase AMP-Activated Catalytic Subunit Alpha 1 *(PRKAA1)*, Protein Kinase AMP-Activated Catalytic Subunit Alpha 1 *(PRKAA1)*, Protein Kinase AMP-Activated Catalytic Subunit Alpha 2 *(PRKAA2)*, Protein Kinase AMP-Activated Non-Catalytic Subunit Beta 1 *(PRKAB1)*. Intercellular Adhesion Molecule 1 *(ICAM1)*, Mitogen-Activated Protein Kinase 11, 12, 13, 14 *(MAPK11, MAPK12, MAPK13, MAPK14)*, Thioredoxin Interacting Protein *(TXNIP)*, Peroxisome Proliferator Activated Receptor Gamma *(PPARG)*, Cocaine- And Amphetamine-Regulated Transcript Protein *(CART)*, Corticotropin Releasing Hormone *(CRH)* Detailed descriptions of these 19 genes associated with ITLN1 expression were obtained from GeneCards^22^ on 2024-May-23 and are listed in the supplemental materials.

## Supplementary Information

Supplementary Information.

Supplementary Tables.

Supplementary Figures.

## Data Availability

We believe all data needed to evaluate the conclusions in the paper are present in the paper and/or the Supplementary Materials. The human RNA-seq data from the LOBB have not been deposited in a public repository due to restrictions by patient consent, but are available from M.B. on request.
