# Supplementary material for: Expression of Intelectin-1, also known as Omentin-1, is related to clinical phenotypes such as overweight, obesity, insulin resistance, and changes after bariatric surgery
Source: Sci Rep. 2024 Sep 27;14:22286. doi: 10.1038/s41598-024-72720-5 (PMC11437189; doi:10.1038/s41598-024-72720-5)
Supplement: Supplementary file 1 — Supplementary Information. [file 41598_2024_72720_MOESM1_ESM.docx]

The following gene list was compiled from ^1^. All gene descriptions were obtained from GeneCards ^2^ on 2024-May-23.

- The C-X-C Motif Chemokine Ligand *(CXCL8)* is a major mediator of the **inflammatory response**. The encoded protein is commonly referred to as interleukin-8 *(IL-8)*
- NLR Family Pyrin Domain 3 *(NLRP3)* is part of a complex that functions as an upstream activator of NF-kappaB signaling, and plays a role in the **regulation of inflammation, the immune response**, and apoptosis.
- Interleukin 6 (IL6) encodes a cytokine that functions in **inflammation** and the maturation of B cells. In addition, the encoded protein has been shown to be an endogenous pyrogen capable of inducing fever in people with autoimmune diseases or infections. The protein is primarily produced at sites of acute and chronic inflammation.
- The Product of Interleukin 1 Beta *(IL1B)* is a member of the interleukin 1 cytokine family. This cytokine is produced by activated macrophages as a proprotein, which is proteolytically processed to its active form by caspase 1 (CASP1/ICE). This cytokine is an important mediator of the **inflammatory response**, and is involved in a variety of cellular activities, including cell proliferation, differentiation, and apoptosis.
- The gene encoding C-C Motif Chemokine Ligand 2 *(CCL2)* This gene is one of several cytokine genes clustered on the q-arm of chromosome 17. Chemokines are a superfamily of secreted proteins involved in immunoregulatory and **inflammatory** processes.
- Tumor Necrosis Factor (TNF) encodes a multifunctional **proinflammatory** cytokine that belongs to the tumor necrosis factor superfamily. This cytokine is mainly secreted by macrophages. It can bind to, and thus functions through its receptors TNFRSF1A/TNFR1 and TNFRSF1B/TNFBR. This cytokine is involved in the regulation of a wide spectrum of biological processes.
- Peroxisome Proliferator Activated Receptor Delta (PPARD) may have a role for this protein in myelination of the corpus callosum, **lipid metabolism,** differentiation, and epidermal cell proliferation.
- Protein Kinase AMP-Activated Catalytic Subunit Alpha 1 (PRKAA1) is the catalytic subunit of the 5'-prime-AMP-activated protein kinase (**AMPK**). AMPK is a cellular energy sensor conserved in all eukaryotic cells. The kinase activity of AMPK is activated by the stimuli that increase the cellular AMP/ATP ratio. AMPK regulates the activities of a number of key metabolic enzymes through phosphorylation. It protects cells from stresses that cause ATP depletion by switching off ATP-consuming biosynthetic pathways.
- Protein Kinase AMP-Activated Catalytic Subunit Alpha 2 (PRKAA2) is another subunit of **AMPK** complex described above.
- Protein Kinase AMP-Activated Non-Catalytic Subunit Beta 1 (PRKAB1) is a non-catalytic subunit of the **AMPK** above.
- Protein Kinase AMP-Activated Catalytic Subunit Alpha 1 (PRKAA1) is the catalytic subunit of the 5'-prime-AMP-activated protein kinase (**AMPK**).
- Intercellular Adhesion Molecule 1 (ICAM1) encodes a cell surface glycoprotein which is typically expressed on endothelial cells and cells of the immune system. It binds to integrins of type CD11a / CD18, or CD11b / CD18 and is also exploited by Rhinovirus as a receptor.
- The Mitogen-Activated Protein Kinase 11 (MAPK11) gene encodes a member of a family of protein kinases that are involved in the integration of biochemical signals for a wide variety of cellular processes, including cell proliferation, differentiation, transcriptional regulation, and development. The encoded protein can be **activated by proinflammatory cytokines and environmental stresses** through phosphorylation by mitogen activated protein kinase kinases (MKKs).
- Mitogen-Activated Protein Kinase 12 (MAPK12) is a member of the mitogen-activated protein kinase family. Activation of this kinase family is a major mechanism for transduction of extracellular signals. Stress-activated protein kinases are one subclass of MAP kinases. The protein encoded by this gene functions as a signal transducer during differentiation of myoblasts to myotubes.
- The Mitogen-Activated Protein Kinase 13 (MAPK13) is a member of the mitogen-activated protein kinase family, described above.
- The Mitogen-Activated Protein Kinase 14 (MAPK14) is a member of the mitogen-activated protein kinase family, described above.
- Thioredoxin Interacting Protein (TXNIP) encodes a thioredoxin-binding protein that is a member of the alpha arrestin protein family. Thioredoxin is a thiol-oxidoreductase that is a major regulator of cellular redox signalling which protects cells from oxidative stress. This protein inhibits the antioxidative function of thioredoxin resulting in the accumulation of reactive oxygen species and cellular stress. This protein also functions as a regulator of cellular metabolism and of endoplasmic reticulum (ER) stress. This protein may also function as a tumour suppressor.
- Peroxisome Proliferator Activated Receptor Gamma (PPARG) encodes a member of the peroxisome proliferator-activated receptor (PPAR) subfamily of nuclear receptors. PPARs form heterodimers with retinoid X receptors (RXRs) and these heterodimers regulate transcription of various genes. Three subtypes of PPARs are known: PPAR-alpha, PPAR-delta, and PPAR-gamma. The protein encoded by this gene is PPAR-gamma and is a regulator of **adipocyte** differentiation. Additionally, PPAR-gamma has been implicated in the pathology of numerous diseases including **obesity**, diabetes, atherosclerosis and cancer. Alternatively spliced transcript variants that encode different isoforms have been described.
- Cocaine- And Amphetamine-Regulated Transcript Protein (CART) encodes a preproprotein that is proteolytically processed to generate multiple biologically active peptides. These **peptides play a role in appetite, energy balance, maintenance of body weight**, reward and addiction, and the stress response. Expression of a similar gene transcript in rodents is upregulated following administration of cocaine and amphetamine. **Mutations in this gene are associated with susceptibility to obesity in humans.**
- Corticotropin Releasing Hormone (CRH) encodes a member of the corticotropin-releasing factor family . In response to stress, this hormone is secreted by the paraventricular nucleus (PVN) of the hypothalamus, binds to corticotropin releasing hormone receptors and stimulates the release of adrenocorticotropic hormone from the pituitary gland. Marked reduction in this protein has been observed in association with Alzheimer's disease. Autosomal recessive hypothalamic corticotropin deficiency has multiple and potentially fatal metabolic consequences including hypoglycemia and hepatitis. In addition to production in the hypothalamus, this protein is also synthesized in peripheral tissues, such as T lymphocytes, and is highly expressed in the placenta. In the placenta it is a marker that determines the length of gestation and the timing of parturition and delivery.

# References

1 Sena CM. Omentin: A Key Player in Glucose Homeostasis, Atheroprotection, and Anti-Inflammatory Potential for Cardiovascular Health in Obesity and Diabetes. *Biomedicines* 2024; **12**: 284.

2 Rebhan M, Chalifa-Caspi V, Prilusky J, Lancet D. GeneCards: integrating information about genes, proteins and diseases. *Trends Genet* 1997; **13**: 163.
