# Supplementary material for: Expression of Intelectin-1, also known as Omentin-1, is related to clinical phenotypes such as overweight, obesity, insulin resistance, and changes after bariatric surgery
Source: Sci Rep. 2024 Sep 27;14:22286. doi: 10.1038/s41598-024-72720-5 (PMC11437189; doi:10.1038/s41598-024-72720-5)
Supplement: Supplementary file 3 — Supplementary Figures. [file 41598_2024_72720_MOESM3_ESM.docx]

**Supplemental Figure 1:** Correlation of VST normalized expression values between ITLN1 and 19 associated genes (reviewed in ^21^) , for subcutaneous adipose tissue (SAT) among the Cross-Sectional-Cohort (CSC). We report FDR corrected Spearman correlations. Further details are provided in Tables 1 and 2 (main text)**.**

**Supplemental Figure 2:** Correlation of VST normalized expression values between ITLN1 and 19 associated genes (reviewed in ^21^) , for visceral adipose tissues (VAT) among the Cross-Sectional-Cohort (CSC). We report FDR corrected Spearman correlations. Further details are provided in Tables 1 and 2 (main text).
